# Pseudotumor in Ceramic-on-Ceramic Total Hip Arthroplasty: A Case Report

**DOI:** 10.7759/cureus.71518

**Published:** 2024-10-15

**Authors:** Danielle Piper, Juliette A Saloway, Lee Jeys, Shakir Hussain

**Affiliations:** 1 Trauma and Orthopedics, Queen Elizabeth Hospital, Birmingham, GBR; 2 Trauma and Orthopedics, University Hospitals Birmingham NHS Foundation Trust, Birmingham, GBR; 3 Orthopedic Surgery, Royal Orthopaedic Hospital, Birmingham, GBR; 4 Trauma and Orthopedics, Royal Orthopaedic Hospital, Birmingham, GBR

**Keywords:** adverse local tissue reaction (altr), aseptic lymphocytic vasculitic associated lesions (alval), ceramic arthroplasty, metallosis, pseudotumour

## Abstract

Pseudotumors are a rare complication of total hip arthroplasty (THA), arising from local soft tissue reactions. These reactions can lead to painful joint effusions and prosthetic loosening, often necessitating revision surgery. Metal-on-metal and metal-on-polyethylene prostheses are particularly prone to this complication due to the accumulation of metal debris from prosthetic wear, which represents a significant drawback. In contrast, ceramic-on-ceramic (CoC) prostheses are considered a superior alternative, offering lower wear rates and avoiding complications related to metal debris. This case report presents a rare instance of pseudotumor formation in a CoC THA. A 57-year-old patient underwent a cementless CoC THA in 2010. Despite developing a pulmonary embolism, the patient experienced no prosthetic-related complications until 2021, when they presented to the Royal Orthopaedic Hospital with concerns about a deep vein thrombosis. MRI and ultrasound scans of the hip revealed a complex collection involving the iliopsoas bursa and a small joint effusion, prompting a biopsy. Histopathology confirmed a pseudotumor with tissue necrosis, macrophages, neutrophils, and ceramic debris. In 2024, the patient underwent revision arthroplasty with excision of the pseudotumor. The original prosthesis was well-fixed, with minimal damage to the ceramic head and acetabular liner, and there were no signs of infection or metallosis. Following the revision, the patient’s pain resolved, and they were satisfied with the outcome of the surgery.

## Introduction

The leading causes of revision total hip arthroplasty (THA) are osteolysis and aseptic loosening, typically resulting from the wear and eventual failure of bearing surfaces [1]. Younger patients, in particular, place higher demands on THA components, with revision rates reported between 18% and 35% at a mean of 15 years after primary THA. Those under 50 and 30 years of age face a two-fold and four-fold increased risk of revision, respectively [2]. This has driven the development of bearing surfaces with lower wear and higher performance characteristics.

Metal-on-metal (MoM) bearing THA and certain hip resurfacing implants demonstrate different wear profiles compared to conventional metal-on-polyethylene THA [3]. However, a significant drawback of MoM bearings is the potential development of aseptic lymphocytic vasculitis-associated lesions (ALVALs), often referred to as “pseudotumors,” caused by adverse local tissue reactions ALTRs to metal debris [4]. These reactions can lead to painful joint effusions, palpable masses, and aseptic loosening of the prosthesis. The Medicines and Healthcare products Regulatory Agency (MHRA) recommends limited use of MoM implants, with long-term postoperative monitoring, including blood cobalt and chromium levels and metal artifact reduction sequence imaging. Revision surgery is indicated when a pseudotumor becomes symptomatic or when aseptic loosening occurs [5].

Ceramic-on-ceramic (CoC) THA has the lowest volumetric wear rate, excellent lubrication, superior scratch resistance, and biocompatibility [6]. These attributes, along with the absence of complications seen with MoM bearings, make CoC a highly appealing option for young or high-demand patients undergoing primary THA. While pseudotumors have been reported in mixed-bearing surfaces, such as ceramic-on-polyethylene [7], only three cases of pseudotumor formation associated with CoC implants exist in the literature. One case involved a fungal infection [8], another a ceramic head fracture with third body wear [9], and the third had no identifiable cause or metallosis [10].

We present the second documented case of pseudotumor formation following CoC THA in the absence of component corrosion or metallosis.

## Case presentation

**Figure 1:**
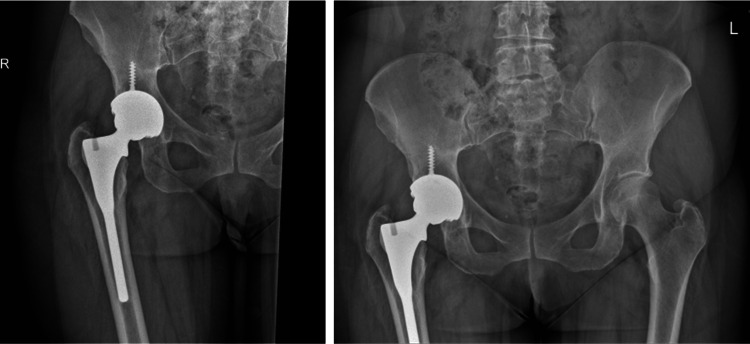
Pre-revision radiographic images. Radiographs demonstrate a right primary THA with a JRI Furlong cementless stem, a 32 mm ceramic head, and a JRI cementless CSF shell with a ceramic liner. THA, total hip arthroplasty

In 2010, a 57-year-old woman underwent a right primary THA for osteoarthritis at a satellite orthopedic surgical center. The patient received a JRI R Furlong cementless stem with a 32 mm ceramic head and a JRI cementless CSF shell with a ceramic liner, as depicted in the radiographic images in Figure 1.

Her postoperative recovery was complicated by a diagnosis of pulmonary embolism (PE), which was successfully treated with warfarin and subsequently long-term apixaban. There were no reported complications consistent with infection.

In October 2021, at the age of 66, the patient presented to our unit with a history of right-sided hip and groin pain. Her medical history was significant for a previous PE, rheumatoid arthritis, and a recently diagnosed deep vein thrombosis (DVT) in the right lower limb. She reported pain around the lateral aspect of her right hip, which progressively extended to the right groin over the following months. This pain was associated with swelling of the right leg, leading to the diagnosis of a DVT.

On examination, her wound had healed well, with no obvious signs of infection. Her range of motion was full, pain-free, and comparable to her contralateral side. Radiographs revealed a well-fixed, well-aligned THA with a ceramic-bearing surface, showing no evidence of lysis or bone loss. Her biochemistry results indicated a CRP level of 18, an erythrocyte sedimentation rate of 44, cobalt ions of <10, and chromium ions of 18, as detailed in Table 1. Cobalt and chromium ion levels were considered in reference to the MHRA guidelines on metal ion levels in patients with MoM hip replacements regarding the occurrence of soft tissue reactions [5].

**Table 1:** Laboratory investigation results for CRP, ESR, and Co and Cr ion levels following ceramic prosthetic implantation. Co, cobalt; Cr, chromium; ESR, erythrocyte sedimentation rate

Laboratory parameter	Patient level	Reference range
CRP	18	0-5 (mg/L)
ESR	44	5-15 (mm/hr)
Co ions	<10	<119 (nmol/L)
Cr ions	18	<134.5 (nmol/L)

A metal artifact reduction protocol MRI revealed a complex collection measuring 7.5 × 3.3 × 10 cm involving the iliopsoas bursa, along with a small joint effusion. Following further swelling in her right lower limb and concerns regarding additional DVT, she underwent another ultrasound (US) scan of her hip after the MRI, which indicated an increase in the size and complexity of the collection, accompanied by newly identified femoral vein compressive distortion.

After a multidisciplinary meeting at the Royal Orthopaedic Hospital, including a review by a senior orthopedic oncology specialist, a decision was made to biopsy the mass to obtain a final diagnosis before proceeding with revision arthroplasty. The biopsy was conducted under US guidance and confirmed the presence of a pseudotumor characterized by tissue necrosis, macrophages, neutrophils, and ceramic debris. After discussing the risks of the procedure, the patient opted for revision arthroplasty and excision of the pseudotumor, conducted as a dual consultant case involving arthroplasty and orthopedic oncology.

**Figure 2:**
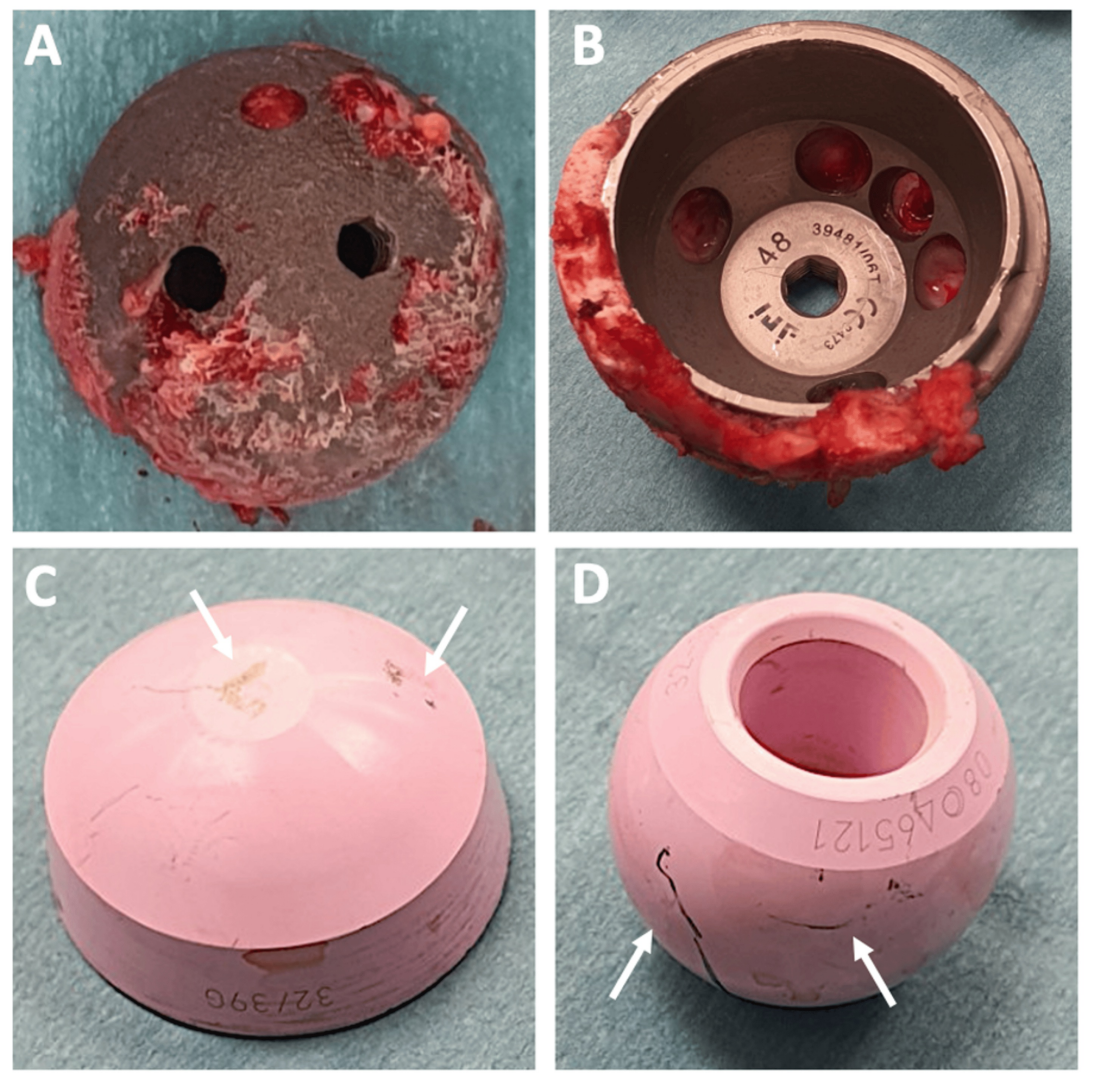
(A) Metal acetabular cup with an intact external surface. (B) Metal acetabular cup with an intact internal surface. (C) Ceramic acetabular liner showing mild damage (white arrows) resulting from surgical hip dislocation during prosthesis removal. (D) Ceramic femoral head showing mild damage (white arrows) caused by surgical hip dislocation during prosthesis removal.

The patient underwent the revision in February 2024. During the procedure, there were no macroscopic signs of infection or metallosis, and the acetabular cup was found to be well-fixed with a 2C defect after explantation. There was no obvious damage to the trunnion. Minimal damage was noted to the ceramic head and the acetabular liner, as shown in Figure 2. The femoral component was well-fixed.

**Figure 3:**
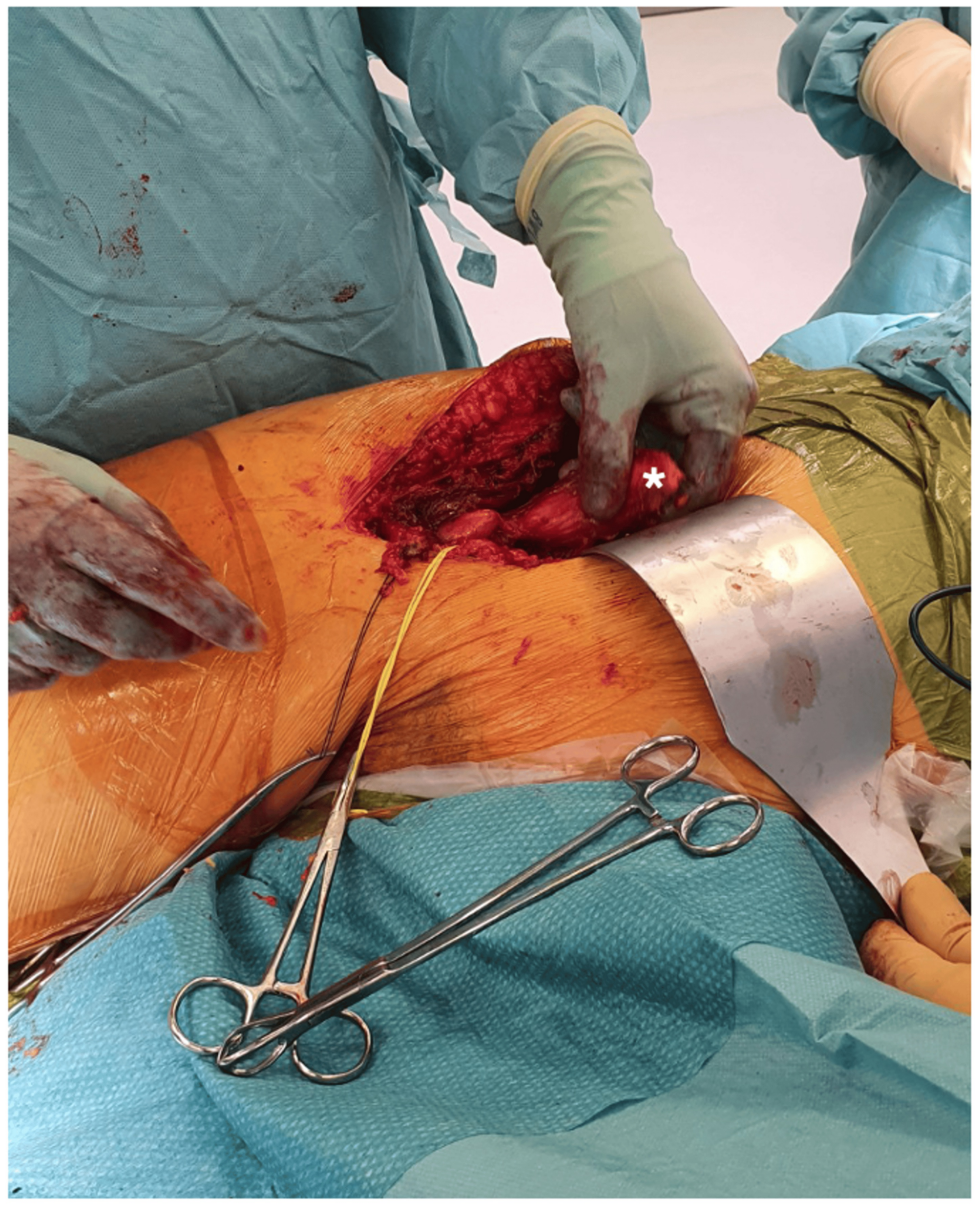
Intraoperative pseudotumor. The pseudotumor is located on the ilioinguinal aspect of the patient’s right thigh, indicated by the asterisk (*).

**Figure 4:**
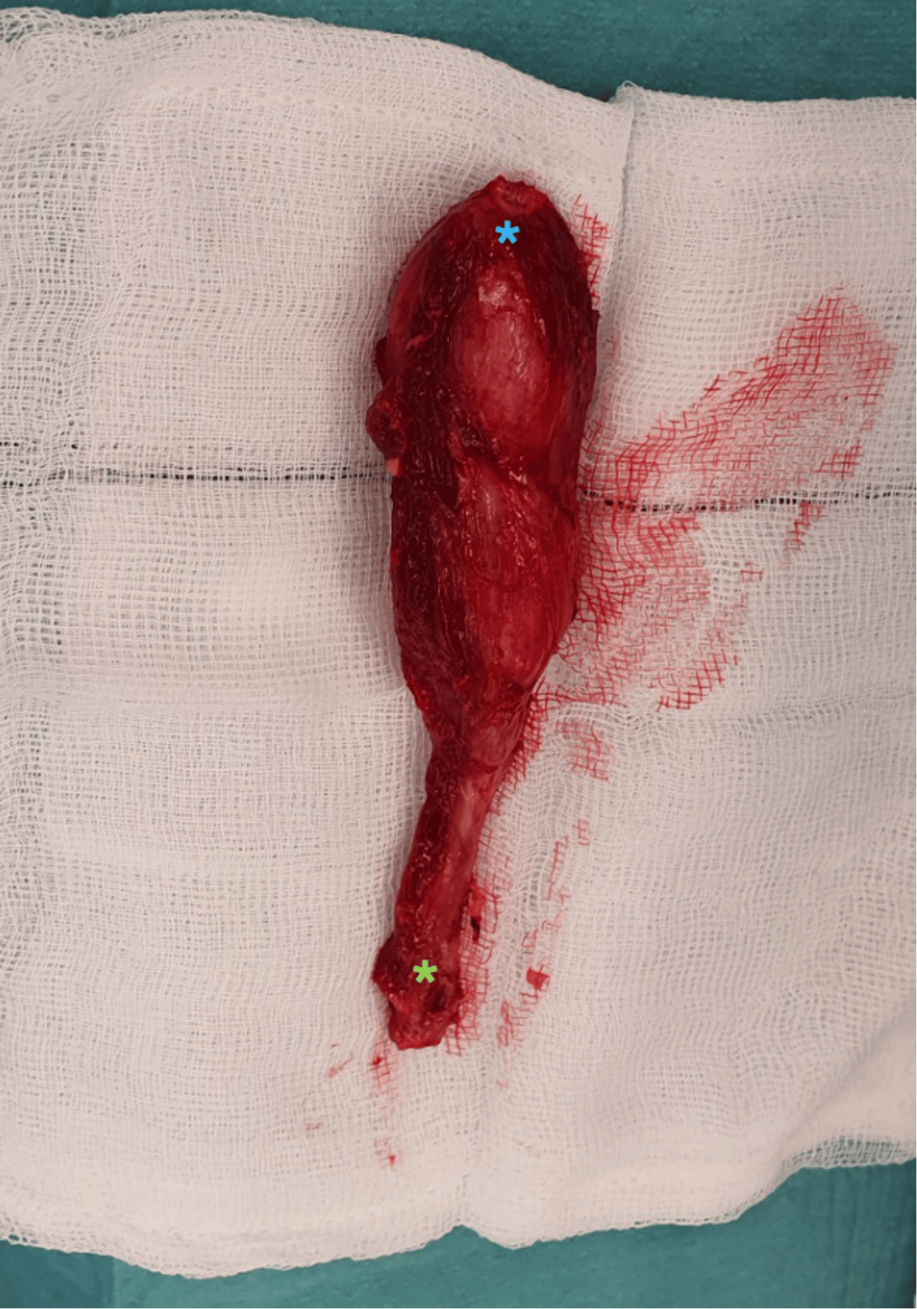
Excised pseudotumor. The pseudotumor was removed from the patient through the ilioinguinal approach. On MRI, it measured 7.5 cm × 3.3 cm × 10 cm. The blue asterisk indicates the proximal pole, while the green asterisk marks the distal pole of the pseudotumor.

The pseudotumor was excised through an ilioinguinal approach and was located in the first compartment, within the psoas and iliacus muscles, extending inferior to the inguinal ligament. The pseudotumor in situ and post-excision are depicted in Figure 3 and Figure 4, respectively. Complete dissection and excision of the macroscopic lesion were successfully achieved.

**Figure 5:**
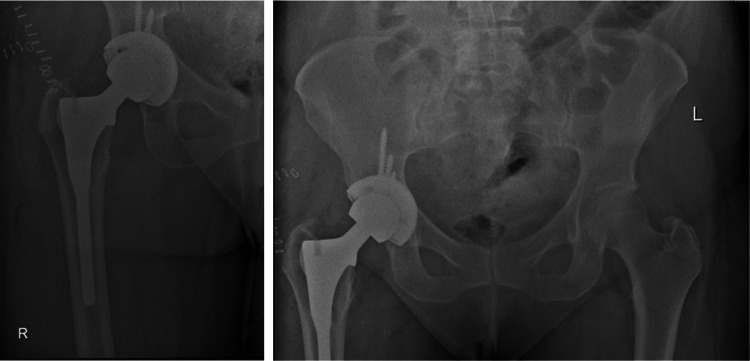
Revision arthroplasty images. Postoperative images depict the inserted REDAPT acetabular cup (Smith & Nephew), R3 liner (Smith & Nephew), and JRI long neck sleeve with a delta ceramic 36 mm head.

After thorough irrigation, a REDAPT acetabular cup (Smith & Nephew) with an R3 liner (Smith & Nephew) and a JRI long neck sleeve with a delta ceramic 36 mm head were implanted, as shown in the patient’s revised radiographic images in Figure 5.

Histopathology results of the excised specimen revealed a macroscopic appearance of irregular, multilobulated tissue. Upon dissection, the cut surface exhibited multiple cream, slightly mucinous soft tissue areas. Microscopically, the sections showed fibrous tissue with underlying infiltration of macrophages accumulating wear debris. Additionally, foamy macrophages, hemosiderin deposition, and multinucleated giant cells were observed. Throughout the lesion, there were areas of large cysts with central necrosis. Overall, the report was consistent with a diagnosis of a pseudotumor.

Postoperatively, the patient’s pain resolved during subsequent clinic reviews, and her leg swelling diminished. The patient expressed satisfaction with the results of her surgery.

## Discussion

ALTR is well-documented and associated with MoM articulations as well as mixed-bearing surfaces in the presence of taper corrosion [11,12]. Although the process of pseudotumor formation remains incompletely understood, current evidence suggests that it may involve a delayed-type hypersensitivity reaction triggered by the release of metal particles due to wear, non-articulating component corrosion, or a combination of these factors.

Histological appearances of ALTR exhibit considerable variation; however, lymphocytic infiltration is theorized to be related to metal debris, delayed hypersensitivity reactions, and the formation of ALVALs. In contrast, macrophage infiltration is associated with aseptic osteolysis resulting from high bearing wear rates [13]. While pseudotumors can be asymptomatic and may not necessitate immediate surgical intervention, their presence is concerning and warrants close clinical and radiographic monitoring. The prevalence of pseudotumors in MoM articulations can range from 36% to 61% in well-functioning hips [14].

Historically, concerns surrounding CoC articulation focused on high femoral head fracture rates, squeaking, and significant third-body wear following revisions. First-generation CoC implants exhibited fracture rates as high as 13.4%, a figure that has since decreased to 0.0010% with modern processing techniques and the introduction of fourth-generation alumina zirconia composites [15]. Squeaking remains a persistent issue, even in new-generation CoC implants, with an incidence of 1-20%. The underlying causes of this phenomenon are unclear, but hypotheses include stripe wear, neck impingement, or changes in fluid-film lubrication [16].

Despite the absence of reports of ALTR in CoC articulations, ceramic debris is not entirely inert; biopsy studies have shown a pro-inflammatory response predominantly involving macrophages when ceramic debris is introduced [17]. However, ceramic generates several thousand times less wear debris compared to polyethylene articulations, potentially resulting in a reduced bio-response and clinical ALTR manifestation. If a ceramic component is incorrectly positioned, its low ductility can make it susceptible to edge loading, potentially leading to abnormally high wear debris generation and provoking a bioresponse consistent with ALTR, although this was not observed in our case. Pseudotumor formation has been previously reported in ceramic-on-polyethylene articulations, but always in conjunction with elevated blood metal ion levels, evidence of trunnion wear, and the presence of metal debris [7].

To our knowledge, there are three documented case reports of pseudotumors associated with CoC articulations. One case involved a concomitant fungal infection in a CoC THA [8], another followed the revision of a CoC THA due to femoral head breakage and was characterized by severe third-body wear and metallosis [9], and the third exhibited the complete absence of clinical metal debris and elevated metal ion levels [10].

Our case report closely resembles the findings of Campbell et al., which described an ALTR pseudotumor in a CoC articulation with no metal debris, component corrosion, implant impingement, or exposed porous coating [10]. This, along with the absence of elevated cobalt and chromium ions, leads us to reasonably conclude that this represents the second documented case of pseudotumor formation due to wear debris in a CoC articulation.

## Conclusions

We report the second case of a pseudotumor in a patient with CoC bearings following total hip replacement. This occurred in the absence of metal debris and without elevated cobalt or chromium ion levels. This case illustrates that, even in the absence of metallosis, ALTRs can manifest in CoC articulations, potentially leading to the formation of a pseudotumor. This finding is significant in the ongoing investigation of fourth-generation CoC arthroplasty and CoC resurfacing. Further research is essential to explore the potential causes and effects of ceramic wear debris contributing to these atypical pseudotumors.
